# Concurrent management of multiple sclerosis and natalizumab-induced hepatitis with ofatumumab: a case report

**DOI:** 10.1007/s10072-024-07614-5

**Published:** 2024-06-11

**Authors:** Shalom Haggiag, Valerio Giannelli, Luca Prosperini, Alessandro Cruciani, Andrea Baiocchini, Serena Ruggieri, Adriano Pellicelli, Claudio Gasperini, Carla Tortorella

**Affiliations:** 1 Department of Neuroscience, Azienda Ospedaliera San Camillo Forlanini, Rome, Italy; 2 Liver Unit, Azienda Ospedaliera San Camillo Forlanini, Rome, Italy; 3 Department of Medicine and Surgery, Unit of Neurology, Neurophysiology, Neurobiology, and Psychiatry, Università Campus Bio-Medico Di Roma, Via Alvaro del Portillo, 21 - 00128 Rome, Italy; 4 Department of Pathology, Azienda Ospedaliera San Camillo Forlanini, Rome, Italy

Dear Sir,

Natalizumab (NAT), a monoclonal antibody targeting α4-integrin, has been a highly effective disease-modifying treatment (DMD) for relapsing forms of multiple sclerosis (MS) since its approval in 2004. NAT works by mitigating inflammation and demyelination through impeding immune cell migration into the central nervous system.

Despite its favourable tolerability profile and lack of hepatic metabolism, phase III trials revealed abnormal liver function in 5% of NAT-treated individuals, with severe cases observed in less than 1%, a trend consistent with long-term extension studies spanning up to 10 years. Hepatotoxicity may occur at any time during treatment, even after initial doses. Similar to other medications, NAT has the potential to induce drug-induced autoimmune hepatitis (DIAIH), a distinct subtype of idiosyncratic drug-induced liver injury (DILI) closely resembling idiopathic autoimmune hepatitis (AIH). Managing DIAIH associated with NAT can be challenging, as discontinuation may trigger a resurgence of MS disease activity. Switching to another rapid-acting, equally effective DMD is essential to prevent short-term disease reactivation. Additionally, certain DMDs like beta-interferons, glatiramer acetate, alemtuzumab, and NAT itself are relatively contraindicated in autoimmune hepatitis due to the risk of exacerbating liver condition. This underscores the importance of selecting an appropriate substitute DMD that addresses both the patient’s MS needs and minimizes the risk of exacerbating liver injury.

This presentation delves into the complex management of an MS patient undergoing NAT treatment, who later develops DIAIH. The patient has been successfully managed with Ofatumumab (OFA), an anti-CD20 monoclonal antibody, for over a year, effectively addressing both MS and hepatitis.

## Clinical case presentation

**Fig. 1:**
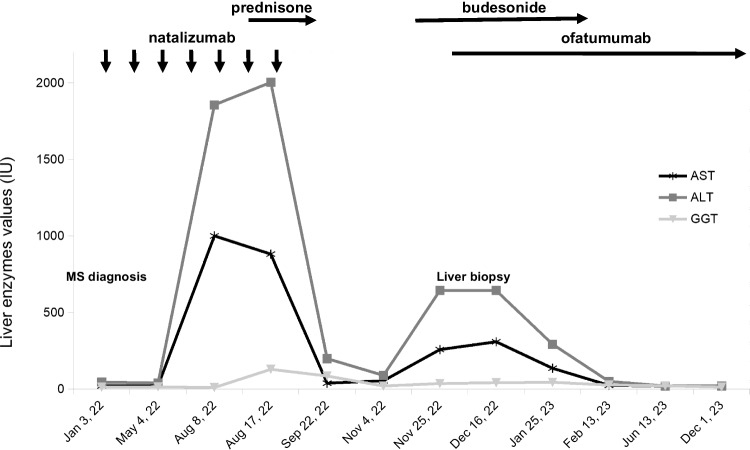
Patient’s clinical course and liver enzyme trend with medication administration

We present the case of a 30-year-old woman diagnosed with MS in February 2022 following optic neuritis. In April 2022, due to a significant lesion burden, she initiated NAT therapy (300 mg every 28 days) as the first DMD. Pre-treatment routine blood tests, including liver function tests (LFT), full blood count (FBC), and infection screenings, were within normal limits. However, after the fourth NAT administration, routine blood tests revealed abnormal LFTs (AST 1000 IU/L, ALT 1857 IU/L, GGT 71 IU/L) and lymphocytosis (WBC 28020 cells/µL, LYM 15930 cells/µL, NEU 9250 cells/µL, EOS 260 cells/µL), with increases in all subpopulations (CD3 + 8540 cells/µL, CD4 + 4896 cells/µL, CD8 + 3247 cells/µL, CD19 + 3625 cells/µL, NK 1481 cells/µL), as well as hypergammaglobulinemia (1.9 g/dL; Fig. 1).

**Fig. 2:**
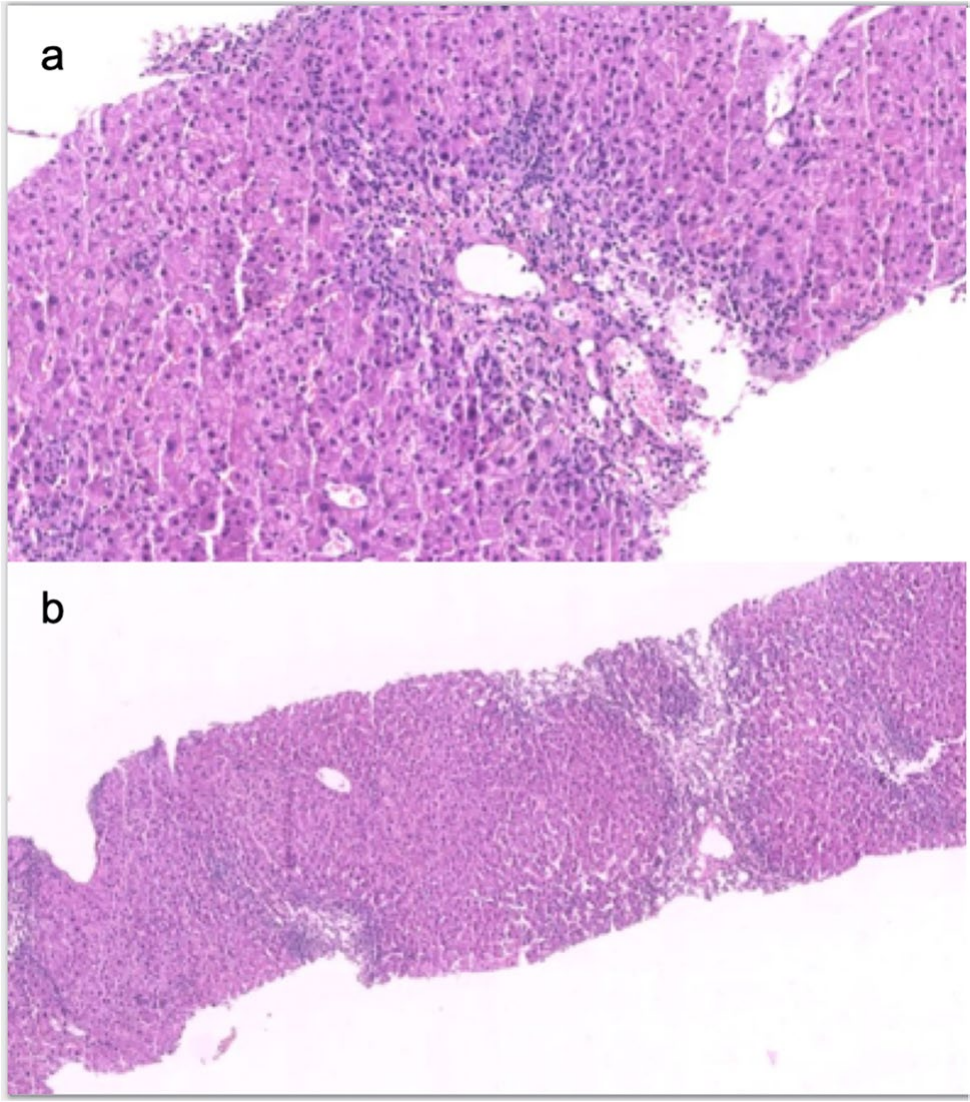
Acute hepatitis with perivenular inflammation with zonal necrosis (**a**) alongside hepatocellular necrosis, accompanied by mild portal inflammation

Although the patient did not display symptoms, abnormal LFT persisted. Alcohol and other drug/supplement intake were excluded, and thorough investigations for infectious, metabolic, and autoimmune causes revealed no irregularities. Abdominal ultrasound, including lymph node stations, showed no abnormalities. Hematological consultation ruled out a lymphoproliferative disorder, confirmed by the absence of clonal B-cell lymphocyte subpopulations. Initially, due to diagnostic uncertainties, the decision was made to continue NAT and initiate prednisone, resulting in a significant reduction in LFT values. However, upon gradual suspension in October, a rebound effect occurred (AST 258 IU/L, ALT 644 IU/L, and GGT 36 IU/L). A liver biopsy revealed extensive perivenular inflammation (Fig. 2a) and extensive periportal necrosis (Fig. 2b), consistent with acute hepatitis. NAT was permanently discontinued and replaced by OFA, accompanied by a low dose of budesonide therapy (for a few months). This led to the thorough normalization of LFT and FBC (Fig. 1). As of April 2024, the patient continues to experience neurological stability with OFA, and LFT remain within the normal range.

## Discussion

Our case highlights autoimmune hepatitis associated with NAT treatment, presenting notable distinctions from previously documented cases in the literature [1–5]. Firstly, the patient’s exclusive exposure to NAT and the occurrence of hepatic relapse following gradual steroid withdrawal, even prior to NAT cessation, suggest it is the primary trigger for the autoimmune response in the liver, minimizing other potential factors. Secondly, despite a significant disruption in LFT, the patient remained asymptomatic throughout the hepatic episode, underscoring the necessity of monitoring liver function from the initial administration, even in the absence of symptoms. Thirdly, the marked lymphocytosis, only partially attributable to NAT itself, which introduced diagnostic uncertainties.

Complete recovery of liver injury is generally observed in cases of DIAIH. However, instances of prolonged injury may occur, necessitating prolonged steroid therapy and/or immunosuppressive therapy. Moreover, relapse following the cessation of corticosteroids for suspected DIAIH should prompt further evaluation for underlying de novo AIH.

In our case, the clinical presentation and histological findings support a diagnosis of DIAIH, and the hepatic relapse following prednisone cessation occurred subsequent to additional exposure to NAT. Nonetheless, the possibility of latent AIH triggered by NAT’s immunological effects cannot be definitively ruled out. Therefore, we opted for a cautious approach. Alongside NAT discontinuation, we initiated OFA combined with low-dose budesonide. Previous cases have been managed with various combinations of steroids and immunosuppressants, including steroids and azathioprine [4], steroids and mycophenolate [2], or steroids alone. One single case has been successfully treated with rituximab [1].

This case report emphasizes, in addition to the importance of a multidisciplinary approach, vigilant liver function test monitoring during NAT therapy, prompt consideration of DIAIH or DILI, and initiation of corticosteroid therapy if acute liver injury occurs. Sustained corticosteroid therapy or appropriate immunosuppression is advisable, particularly when liver damage is consistent. The successful use of an anti-CD20 monoclonal antibody in our case may provide insights for similar cases.

## Data Availability

Additional data are available upon request from the corresponding author.
