# Correction to: Concurrent management of multiple sclerosis and natalizumab‑induced hepatitis with ofatumumab: a case report

**DOI:** 10.1007/s10072-024-07694-3

**Published:** 2024-07-17

**Authors:** Shalom Haggiag, Valerio Giannelli, Luca Prosperini, Alessandro Cruciani, Andrea Baiocchini, Serena Ruggieri, Adriano Pellicelli, Claudio Gasperini, Carla Tortorella

**Affiliations:** 1 Department of Neuroscience, Azienda Ospedaliera San Camillo Forlanini, Rome, Italy; 2 Liver Unit, Azienda Ospedaliera San Camillo Forlanini, Rome, Italy; 3 Department of Medicine and Surgery, Unit of Neurology, Neurophysiology, Neurobiology, and Psychiatry, Università Campus Bio-Medico Di Roma, Via Alvaro del Portillo, Rome, 21 ‑ 00128 Italy; 4 Department of Pathology, Azienda Ospedaliera San Camillo Forlanini, Rome, Italy

**Correction to: Neurological Sciences (2024)**

10.1007/s10072-024-07614-5

The article “Concurrent management of multiple sclerosis and natalizumab‑induced hepatitis with ofatumumab: a case report”, written by Shalom Haggiag, Valerio Giannelli, Luca Prosperini, Alessandro Cruciani, Andrea Baiocchini, Serena Ruggieri, Adriano Pellicelli, Claudio Gasperini and Carla Tortorella, was originally published electronically on the publisher’s internet portal on 11 July 2024 without open access. With the author(s)’ decision to opt for Open Choice the copyright of the article changed on 12 July 2024 to © The Author(s) 2024 and the article is forthwith distributed under a Creative Commons Attribution 4.0 International License, which permits use, sharing, adaptation, distribution and reproduction in any medium or format, as long as you give appropriate credit to the original author(s) and the source, provide a link to the Creative Commons licence, and indicate if changes were made. The images or other third party material in this article are included in the article’s Creative Commons licence, unless indicated otherwise in a credit line to the material. If material is not included in the article’s Creative Commons licence and your intended use is not permitted by statutory regulation or exceeds the permitted use, you will need to obtain permission directly from the copyright holder. To view a copy of this licence, visit http://creativecommons.org/licenses/by/4.0.

The original article has been corrected.

